# Reproductive parameters and cub survival of brown bears in the Rusha area of the Shiretoko Peninsula, Hokkaido, Japan

**DOI:** 10.1371/journal.pone.0176251

**Published:** 2017-04-25

**Authors:** Michito Shimozuru, Masami Yamanaka, Masanao Nakanishi, Jun Moriwaki, Fumihiko Mori, Masakatsu Tsujino, Yuri Shirane, Tsuyoshi Ishinazaka, Shinsuke Kasai, Takane Nose, Yasushi Masuda, Toshio Tsubota

**Affiliations:** 1 Department of Environmental Veterinary Science, Graduate School of Veterinary Medicine, Hokkaido University, Sapporo, Japan; 2 Shiretoko Museum, Shari, Japan; 3 Shiretoko Nature Foundation, Shari, Japan; Université de Sherbrooke, CANADA

## Abstract

Knowing the reproductive characteristics of a species is essential for the appropriate conservation and management of wildlife. In this study, we investigated the demographic parameters, including age of primiparity, litter size, inter-birth interval, reproductive rate, and cub survival rate, of Hokkaido brown bears (*Ursus arctos yesoensis*) in the Rusha area on the Shiretoko Peninsula, Hokkaido, Japan, based on a long-term, individual-based monitoring survey. A total of 15 philopatric females were observed nearly every year from 2006 to 2016, and these observations were used to estimate reproductive parameters. The mean age of primiparity was 5.3 ± 0.2 (SE) years (*n* = 7, 95% CI = 5.0–5.6). We observed 81 cubs in 46 litters from 15 bears. Litter size ranged from one to three cubs, and averaged 1.76 ± 0.08 (SE) cubs/litter (95% CI = 1.61–1.91). Inter-birth intervals ranged from 1 to 4 years, and the mean value was estimated as 2.43 (95% CI = 2.16–2.76) and 2.53 (95% CI = 2.26–2.85) years in all litters and in litters that survived at least their first year, respectively. The reproductive rate was estimated from 0.70 to 0.76 young born/year/reproductive adult female, depending on the method of calculation. The cub survival rate between 0.5 and 1.5 years ranged from 60 to 73%. Most cub disappearances occurred in July and August, suggesting that cub mortality is mainly due to poor nutrition in the summer. All reproductive parameters observed in the Rusha area on the Shiretoko Peninsula fell within the range reported in Europe and North America, and were among the lowest or shortest age of primiparity, litter size, and inter-birth intervals, and ranked at a high level for reproductive rate.

## Introduction

Conservation and management of large carnivores is highly important and challenging, not only due to their potential threat to human livelihood, but also due to their vulnerability to extinction and ability to structure ecosystems [1]. For effective wildlife conservation and management, evaluating demographics is essential, which requires an understanding of species’ reproductive characteristics. The brown bear (*Ursus arctos*), a representative species of carnivores, is a species that is highly adapted to the global environment, with a wide distribution throughout the Northern Hemisphere, including Europe, Asia, and North America. The brown bear shows a typical *K*-strategy, characterized by low reproductive output and long life expectancy [2]. Brown bear reproductive characteristics, including age of primiparity, litter size, inter-birth interval, reproductive rate, and cub survival rate, are well described for western Europe and North America populations [3]. In Scandinavia and the Greater Yellowstone Ecosystem in the USA, for example, large-scale surveys, based on long-term monitoring, have been conducted, and have clarified regional differences and temporal changes in reproductive performance [4, 5]. In contrast, in Asia, including Japan, only a few studies concerning their reproductive characteristics have been conducted [6–8].

In Japan, brown bears are found only in Hokkaido, the northernmost island of Japan. Before intensive land development began in the late 19^th^ century, bears inhabited areas throughout Hokkaido, from coastal to forested areas. Bears continue to range over 60% of the island; however, their habitat has been fragmented by deforestation and extermination, and some local populations are threatened with extinction [9]. In addition, human-bear conflicts, including agricultural crop depredation and intrusion into human residential areas, have become a serious problem throughout Hokkaido [9]. To reduce the conflicts and potential injuries to humans, 300–800 bears have been killed annually over the past decade, mainly for management purposes and partially by legal hunting; although we know very little about the abundance and dynamics of this population. For proper conservation and management of the brown bear population in Hokkaido, it is necessary to accumulate scientific information concerning the ecology of Hokkaido brown bears. However, most of the current knowledge about brown bear reproductive biology in Japan is not based on long-term, individual-based monitoring surveys, but rather through less directed methods, including uterine and ovarian examinations of harvested bears [8, 10–12]. This anatomical analysis has advantages in that a large number of samples can be used to calculate reproductive parameters, such as implantation rate (i.e., the number of placental scars); however, it is unsuitable for estimating other parameters, including inter-birth intervals, reproductive rate, and cub survival rate.

**Fig 1:**
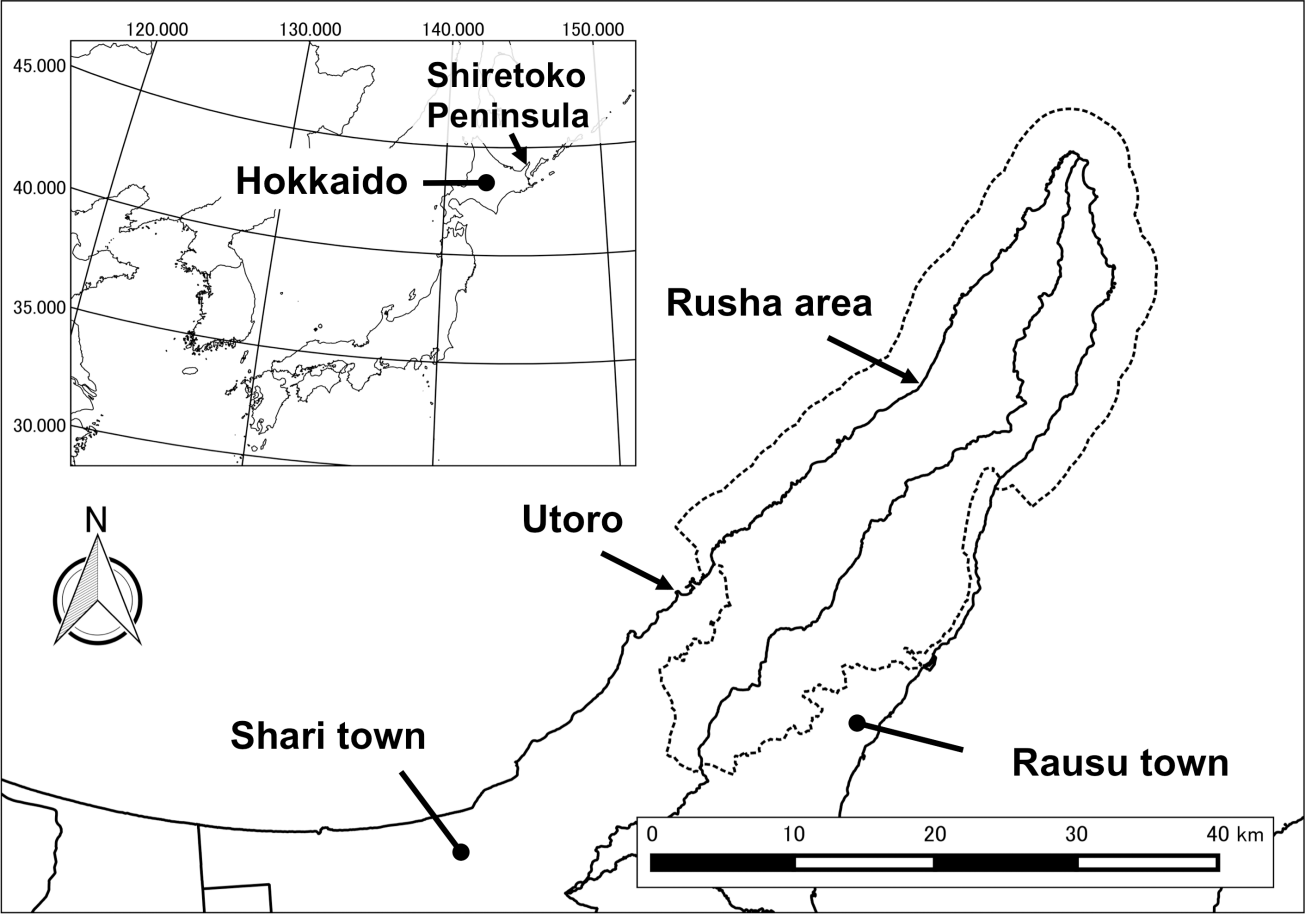
Map of the Shiretoko Peninsula, eastern Hokkaido, Japan. The dotted line indicates the UNESCO World Natural Heritage Site. This figure was developed using National Numerical Land Information (Administrative Zones, World Natural Heritage), edited by the author. Publication of the figure under a CC BY license was permitted by National Spatial Planning and Regional Policy Bureau, MLIT of Japan, copyright 1974–2017.

The Shiretoko Peninsula, located in eastern Hokkaido, has one of the highest bear densities in Japan. The minimum population size was estimated at 200 in 1135 km^2^ including the towns of Shari and Rausu (Fig 1), and the estimated density is ≥ 17.6 bears/100 km^2^ [13]. An area from the middle of the peninsula to the tip, covering 386 km^2^, has been designated as a national park, and an area including the national park and the surrounding continental and marine area, covering 711 km^2^, was designated as an UNESCO World Natural Heritage Site in July 2005, because of its remarkable ecosystems and biodiversity. Annual home ranges of adult females in the Shiretoko Peninsula are typically less than 30 km^2^, whereas those of adult males are expected to exceed more than 400 km^2^ [13]. Near the tip of the peninsula, there is a special wildlife protection area, referred to as the Rusha area, where brown bears live at a high density. The Rusha area is located at the mouths of three rivers where the spawning migrations of a large number of pink salmon (*Oncorhynchus gorbuscha*) and chum salmon (*O*. *keta*) occur from late August to early October [14]. Brown bears utilize this area for food during their active period from April to December, and aggregate especially during the salmon spawning season. Public access is not allowed without permission, and there are no human residents except for one fishermen’s settlement used from May to November. Additionally, because fishermen have not excluded bears from this area over the last few decades, bears have become habituated to the existence of humans, which enables direct observation at close range. Therefore, this area is one of a handful of places, such as the McNeil River Game Sanctuary [15], where long-term monitoring of identifiable bears is possible. In this study, we recorded the reproductive histories of female brown bears in Hokkaido to estimate age of primiparity, litter size, inter-birth interval, reproductive rate, and cub survival rate.

## Materials and methods

### Ethics statement

All procedures were conducted in accordance with the Guidelines for Animal Care and Use of Hokkaido University, and were approved by the Animal Care and Use Committee of the Graduate School of Veterinary Medicine, Hokkaido University (Permit Number: JU1152 and 15009).

### Study area

This study took place in the Rusha area (44°11′N, 145°11′E) of the Shiretoko Peninsula, eastern Hokkaido, Japan (Fig 1). The climate is subarctic, with a mean temperature of 6.6°C and mean annual precipitation of 1,353 mm from 2006 to 2016 at Utoro (Fig 1; 44°03′ N, 144°59′ E), a gateway community near the national park. The terrestrial vegetation up to 700 m elevation is characterized by mixed forests of coniferous and deciduous broad-leaved trees, e.g., *Abies sachalinensis*, *Picea jezoensis*, *Picea glehnii*, *Quercus crispula*, *Acer mono*, and *Alnus hirsuta*. Subalpine and alpine regions are mainly covered by *Betula ermani* and *Pinus pumila*, respectively.

### Monitoring of bears

Occasional surveys (≤ 5 days/year), mainly for the visual counts of bears and their respective offspring, have been conducted since the late 1990s [16]. The data analysis for reproductive parameters commenced in 2006, when the annual number of research days was increased to 7 days. Then, in parallel with an increase in research days, the surveys became periodic (≥ 1 days/two weeks) in 2011, which made it possible to follow annual changes in reproductive activity more closely. The annual number of research days was less than 10 days in 2006–2007, increased to around 20 days in 2008–2010, then 39–60 days in 2011–2016 (Table 1). Additionally, in 2010–2016, we analyzed videos that were recorded by a broadcasting company for a nature program. In total, in 2006–2016, field research was conducted for 363 days, and recorded videos from an additional 96 days were used for the analysis. In general, bears begin hibernation in November–December, and pregnant females give birth to cubs between late January and early February [17]. They emerge from their dens between March and May. The survey was primarily conducted during active periods, except for the first month and last few weeks, when the area was inaccessible by car due to snow.

**Table 1:** Number of research days from 2006 to 2016.

	May	Jun	Jul	Aug	Sep	Oct	Nov	Dec	Total
2006	0	1	1	1	2	1	1	0	7
2007	1	0	1	0	0	3	3	0	8
2008	0	1	2	1	0	11	2	1	18
2009	0	6	6	6	3	4	0	0	25
2010	0	0	6	0	9 (3)	2	5	0	22 (3)
2011	0	1	12	8	7	10 (6)	7 (6)	0	45 (12)
2012	6 (6)	13 (12)	14 (6)	10 (5)	7 (1)	3	7 (3)	0	60 (33)
2013	3	14	9 (3)	8	5	3	4	0	46 (3)
2014	0 (1)	8 (5)	6 (8)	12	8	5	6 (6)	0	45 (20)
2015	0	10 (1)	8 (8)	12 (2)	7 (6)	6	5	0	48 (17)
2016	0 (8)	7	9	10	7	5	1	0	39 (8)
Total	10 (15)	61 (18)	74 (25)	68 (7)	55 (10)	53 (6)	41 (15)	1	363 (96)

Numbers in parentheses indicate the number of days recorded on videotape and analyzed thereafter by the researchers.

Field teams of two to five people patrolled the area by car from approximately 10 a.m. to 5 p.m. This area is a narrow estuarine coast, stretching south to north for approximately 3 km in length. While driving back and forth, the field team waited for bears to emerge from the vegetation on the mountainside. When bears appeared, we followed individuals, staying at a distance of about 20–100 m. The time of the appearance and the individual’s status, e.g., sex (if discriminable), estimated age class (i.e., adults, independent young, dependent young; defined later), and the presence or absence and the number of offspring were recorded. Individual bears were temporarily identified by the field staff according to their appearance (detailed below), and close-up photographs were taken from multiple angles for later confirmation by multiple researchers. The field team in each survey included at least one of three core members who had a long experience with the bears inhabiting the area. Most bears appeared repeatedly throughout the surveillance period, which allowed us to recognize individuals that do not have any artificial markings, e.g., ear tags. Individual recognition was done mainly by morphology, which has been used in other brown bear studies [7, 15, 18, 19]. The following factors were used in identifying individuals:

1. Chest markings: Over half of the bears had characteristic white chest markings. The size and the shapes were variable; some had a large bib-like marking, some had a small point-like marking on the shoulder, and in most cases, the shapes were asymmetrical (S2 Fig). Individual patterns did not change seasonally or through years (For an example, see S3B Fig), so markings, if they existed, could be used as the most decisive factor in the identification of individual bears, similar to other brown bear study [7] and other bear species [20].
2. Color variation and facial characteristics: Hair color varied among individuals; black, light brown, dark brown, silvertip, and blond. Some had characteristic color patterns, e.g., one had lighter coloration on the side as compared to other body areas. Also, close-up photographs enabled us to discriminate visually the bears by their facial characteristics (S1 Fig). Some facial characteristics did not change seasonally or annually, which could be used for individual identification (For an example, see S3A Fig).
3. Size: Brown bears are sexually dimorphic in size, which makes it possible to differentiate matured males (especially males ≥ 7 years old [17]) from the others. The pattern of urination, and the external genitalia and long vulval hair, if observed, also aided in sex determination.
4. Ear tags and GPS collar: In some cases, bears with ear tags and/or a GPS-collar were observed in the Rusha area. These bears (one adult female and two adult males) were captured for research purposes (before or during the study period) outside the Rusha area in the Shiretoko Peninsula. Additionally, eight female bears (six adult females and two independent young; S2 Table) were captured in the Rusha area from 2013 to 2015 for behavioral tracking by GPS collar. Bears were immobilized by dart injection using a CO_2_ injection rifle (Model J.M.ST; Dan-Inject, Børkop, Denmark) with an intramuscular administration of zolazepam HCL and tiletamine HCL cocktail (Zoletil^®^, Virbac, Carros, France) and medetomidine HCL (Domitor^®^, Zenoaq, Japan). The bears were released after they were ear-tagged and a GPS collar was attached.
5. Genetic analysis: A genetic analysis has been underway since 2009, and partly supported the individual monitoring. Briefly, genetic samples were collected from feces, hairs by using hair-traps, and skin tissues collected by dart-biopsy, and the genotype and sex were determined by microsatellite analysis [21, 22] and PCR targeting the amelogenin gene [23], respectively. The genetic analysis verified individual identification and blood relationships between mothers and their offspring. In addition, this genotyping technique was especially useful for confirming that young bears were alive after separation from their mothers. For more details, see supplemental information in S1 Text.

### Age and sex structure of the bears observed in the Rusha area

The age and sex composition of the bears observed in the Rusha area has been followed since 2008. Due to the limited number of research days, data from 2006 and 2007 were not included in the analysis. We calculated the minimum and maximum number of annually observed bears for each age class. For this analysis, we excluded ambiguous observations in which a bear was not clearly recorded by the observers or by photograph, e.g., the target was too far away from the observers, moved away into the bush, or was poorly visible due to light conditions. The minimum number included only bears that were definitely different from each other and free from the possibility of overlap. The maximum number included additional bears that appeared to be different, but were hard to exclude from possibly overlapping with the bears included in the minimum number. The bears were classified into four age and sex classes, including adult females, defined as females ≥ 4 years old (minimum age of first parturition in Hokkaido [6]), adult males, defined as males ≥ 4 years old, independent young (1–3 years), and dependent young (0–2 years). Female age class was estimated according to the existence of birth experience and body size, and females that had given birth to cubs in a given year were assumed to be ≥ 4 years old. Male age class was estimated according to body size, and males that consorted with adult females in the mating season were assumed to be ≥ 4 years old. In some cases, age class was estimated using an analysis of cementum annuli present in the teeth of bears captured or killed outside the Rusha area (one adult female and two adult males were captured for research purposes; two adult males and three independent young males were killed for management purposes or by hunting). Because the sex of young bears is difficult to determine from appearance, they were not classified by sex. Young that were with their mother in the spring but separated after the mating season in the same year were categorized as independent young. Final confirmation of individual identification and the classification of observed bears were performed with the consensus by multiple researchers.

### Estimation of reproductive parameters

Reproductive parameters were estimated by focusing on 15 female bears that could be easily identified and were frequently observed in the area throughout the surveillance period. In the year the bears were first observed since 2006, nine bears (bear ID: KR, WK, BE, RI, WM, LI, DC, KB and KS) were obviously mature (i.e., ≥ 4 years); two bears (DR and PK) were under 4 years old; and four bears (HC, GB, GI, and BK) were born after 2006 (Table 2). The ages of four of the bears as of 2006 were confirmed by prior survey (LI and RI were born in 1996 and 2002, respectively, and DR and PK were born in 2004). These four bears were firstly observed as cubs (defined as 0 years old) with their mothers (KB, GH, BE, and DC, for LI, RI, DR, and PK, respectively. GH was not observed during the study period, and thus was not included in the analysis), and observed annually in the following 5 years (LI) or 4 out of the following 5 years (RI, DR, and PK).

**Table 2:** Reproductive histories of female brown bears in the Rusha area, Shiretoko Peninsula, Hokkaido, Japan, 2006–2016.

Bear ID	Year										
	2006	2007	2008	2009	2010	2011	2012	2013	2014	2015	2016
KR	S	C (2)	SW	C (2)	SW	C (2)	SW	C (2)	SW	C (2)*	S
WK	S	C (1)*	S	C (1)*	S	C (2)	Y (2)	SW	C (3)	Y (2)	SW
DR	sub	n.o.	S	C (1)*	S	C (2)	Y (2)	SW	C (2)	SW	S
BE	S	C (2)	SW	C (2)	SW	C (2)	Y (2)	SW	C (3)	SW^§^	
PK	sub	n.o.	S	C (3)	S	C (1)	Y (1)*^§^				
RI	S	n.o.	S	S	C (2)	Y (2)	T (1)^¶^	SW	C (2)	S	C (1)
WM	C (2)	Y (2)	SW	S	C (2)	Y (2)	SW	C (1)*	C (2)	SW	S
LI	C (1)	S	C (2)	SW	C (2)	SW	C (1)*	C (2)	SW	C (2)	Y (1)
DC	n.o.	n.o.	n.o.	SW^#^	C (2)	SW	C (2)	SW	C (2)	Y (2)	SW
KB	C (2)	n.o.	C (1)	SW	C (2)	SW	C (2)	S	S	S	S
KS						S	C (1)	S	C (1)	SW	C (1)
HC		birth	sub	sub	sub	S	C (1)*	S	C (2)	SW	C (1)
GB				birth	sub	sub	sub	S	S	C (2)*^§^	
GI					birth	sub	sub	sub	S	C (2)	S
BK						birth	sub	sub	sub	S	S

S, Solitary; SW, Solitary after successful weaning of offspring; C, with cubs (number); Y, with yearlings (number); T, with 2-year-old offspring (number); n.o., not observed; sub, subadult (1–3 years old)

C* and Y* indicate that the bear lost all cubs or yearlings, respectively, in the year.

^§^These females died after the successful weaning of the yearlings (BE) or after they lost all cubs (GB) or the yearling (PK).

^#^The bear was solitary when observed in July but was accompanied by one offspring of unknown age in August and September.

^¶^One of the two offspring was weaned, and the other remained with the mother in the year.

#### Age of primiparity

We determined the age of primiparity of females whose birth year was known. Calculation of the average age of primiparity, using only bears whose first litters were observed, gave low-biased estimates, when late-matured bears were censored before giving birth. To reduce bias, nulliparous females (≥ 5 years) that did not produce a litter by the end of the study were treated as having produced in the following year according to the previous studies [7, 24], because the mean age of primiparity among the five females that produced cubs during the study period was < 6 years. The 95% confidence intervals were generated from bootstrapping (2,000 re-samplings) by using Microsoft Excel software for Mac.

#### Litter size

To determine the mean litter size, we used observed litters as the sample unit. We included only litters that were first observed during their cub year from May to November. The 95% confidence intervals were generated from bootstrapping (2,000 re-samplings) by using Microsoft Excel software for Mac.

#### Inter-birth intervals

To determine the inter-birth interval, we followed the procedure used by Garshelis et al [24, 25]. We used each litter interval as the sample unit. This method generates an unbiased estimate by using all data, whether the interval is completed (i.e., closed inter-birth interval) or not. We tallied the number of years from production of one litter to production of the next litter. If a record ended during a period between litters (i.e., right censored), we used the data up to that point. We excluded a record that was started during an interval between births (i.e., left censored) from the analysis. The inter-birth intervals were calculated in two ways, by using all litters, and by using only litters that survived at least their first year. The 95% confidence intervals of the inter-birth intervals were generated from bootstrapping (2,000 re-samplings) based on data set presented in Table 5 by using Microsoft Excel software for Mac.

#### Reproductive rate

To calculate reproductive rate (young born/year/reproductive adult female), we used two methods: the first based on litter size and inter-birth intervals, i.e., dividing the mean litter size by the mean litter interval (method 3 of [26]); and the second dividing the number of cubs produced by the number of adult bear-years observed from 2006 to 2016 (method 1 of [26]). The second approach was further divided into two conditions. In the first case, we included all adult bears ≥ 4 years, the potential age of first parturition of Hokkaido brown bears [6], and excluded the records if the bear was < 4 years old. In the second case, we included bears ≥ 5 years, according to the age of average female primiparity in brown bears (the minimum value is 5.2 in central Sweden [3, 4]), and excluded the records if the bear was < 5 years old. Out of 15 females monitored in this study, the birth year was known for 8 bears, and another 5 bears were known to be ≥ 5 years as of 2006, due to the birth experience observation in the past survey. However, for two bears (bear WK and KS; Table 2), the age at first record was not known, and it was possible that they were 4 years old in the year. These cases were included or excluded in bear-years ≥ 5 years old, as the minimum and maximum values of the reproductive rate for bears ≥ 5 years old, respectively. The 95% confidence intervals were generated from bootstrapping (2,000 re-samplings) by using Microsoft Excel software for Mac.

### Estimation of cub survival rates

We estimated the first-year (from approximately 0.5 to 1.5 years old) survival rates by focusing on cubs produced by the above 15 females from 2006 to 2015. The first-year survival was determined by the cub being observed in the following year, or later. When a cub disappeared from its mother, the cub was regarded as dead, similar to other studies [4, 27, 28]. In brown bears, most mother-offspring separation occurs before and during the mating season (May to June) [29]. Our study did not always cover the period from the emergence from the den with the mother to the onset of the mating season; therefore the disappearance of a yearling from its mother indicated two possibilities: death or weaning. In our analysis, the former was used as the worst-case scenario, and the latter as best-case scenario. The first-year survival rate was calculated by dividing the number of cubs surviving to the next year by the total number of cubs. In addition, to assess time course changes in cub survival rate in the first year, we calculated the survival rate bimonthly from June to November by using the Kaplan-Meier technique [30]. In this approach, cubs were censored once the family disappeared or if the cub was never observed afterward. The data on three cubs born in 2016 were included in this analysis.

## Results and discussion

### Age and sex structure of the bears observed in the Rusha area

The number of bears observed annually for each sex and age class is presented in Table 3. We do not deny the potential presence of misclassifications, as age and/or sex determination in some cases was based solely on body size. However, it seems plausible that these had a limited influence on the overall result. In 89–94% and 82–84% of cases of bears categorized as adult females and adult males, respectively (the smallest and the highest values were calculated based on maximum and minimum numbers of annually observed bears, respectively), both sex and age class were determined not only by body size, but also in more reliable ways, such as the existence of birth experience, consorting behavior between male and female in the mating season, analysis of cementum annuli present in teeth when captured or killed, and a combination of decisive factors for age (i.e., birth record) and for sex (i.e., visual confirmation of the external genitalia, pattern of urination, and genetic analysis). When limited to sex confirmation, the percentage increased to 99–100% and 89–92% for adult females and adult males, respectively. Except for bears which appeared immature and were estimated, at maximum, as 2 years old, there were only four cases in which both sex and age class were uncertain (one and three cases were categorized in adult females and independent young, respectively, based on body size). Additionally, there were eight cases in which the sex was determined as female by visual confirmation or genetic analysis (but the age class was uncertain) and were included in independent young, based on body size. In independent young, there were no cases in which the sex was determined as male and age class was uncertain. On average, from 2008 to 2016, approximately 40 individuals, including 15 adult females, 3 adult males, 9 independent young, and 13 dependent young, were observed annually in the Rusha area. The adult sex ratio was strongly biased toward females; the female to male ratio was approximately 5:1. This disequilibrium is attributed mainly to male bears’ high vigilance against humans, because adult males that migrate from different areas do not become acclimated to the presence of humans. In fact, opportunities for the observation of adult males were rare compared to those for adult females, even in the salmon-spawning season, and dominance by adult males over salmon resources was not observed (at least in the daytime). In this study, we were able to follow 15 philopatric females nearly every year, which we used to estimate reproductive parameters. Compared with broad-scale individual-based monitoring studies conducted in western Europe [2, 4] and North America [5, 31, 32], the current study demonstrated several advantages in that the research could be conducted in a small area without capturing bears, thereby the continuity could be maintained with less effort in a low-cost manner. However, we also note that there are some limitations in the current method. First, it is still unclear if the monitored females are representative of the brown bear population in the Shiretoko Peninsula. Second, as described above, the sex and age class classification is difficult in some cases where unfamiliar middle-sized bears, between young to adult female body sizes, appeared opportunistically.

**Table 3:** Age and sex structure of the brown bears observed in the Rusha area, Shiretoko Peninsula, Hokkaido, Japan, 2008–2016.

Year	Adult female^*^	Adult male^§^	Independent young^#^	Dependent young^¶^	Total
2008	11–13	2–3	9–12	7	29–35
2009	15–16	3	8–10	13	39–42
2010	10	1	7–9	10	28–30
2011	14	4	14	17	49
2012	19–21	3	8–10	18	48–52
2013	14–16	5–6	9–10	5	33–37
2014	20–21	3–4	7	21	50–52
2015	18–19	1	6	19	44–45
2016	14–15	3	5–6	5	27–29
Average	15.0–16.1	2.8–3.1	8.1–9.3	12.8	38.6–41.2

^*^Female bears ≥ 4 years old

^§^Male bears ≥ 4 years old

^#^Independent subadult bears (1–3 years old)

^¶^cubs, yearlings, and two-years-old bears dependent on their mothers

The range represents minimum and maximum counts based on the possible overlap.

### Estimation of reproductive parameters

#### Age of primiparity

Out of seven females with reliable age estimates that were nulliparous when first observed (DR, PK, RI, HC, GB, GI and BK), five females produced litters during the observation period (Tables 2 and 4). One bear (RI) was not observed at the age of five in 2007, and thereby was excluded from the analysis of age of primiparity. The age of first litter production was 5 years in four females (DR, PK, HC, and GI) and 6 years in one female (GB). One adult bear (LI) was known to have produced two cubs at the age of five according to a previous survey [16], which was included in the analysis. One female (BK, 5 years old in 2016) was censored before giving birth, and treated as having produced at 6 years old. Taken together, the mean age of primiparity was 5.3 ± 0.2 (SE) years (95% CI = 5.0–5.6). In the cases of first reproduction in this study, the average litter size was 1.83 cubs/litter, and the cub survival rate ranged from 18% (worst-case scenario) to 36% (best-case scenario). The estimated survival rate in this study was potentially high, because it was known that primiparous bears tend to lose cubs in the pre-mating season, from birth to shortly after leaving dens, and during the mating season [33]. It has been reported that primiparous females have smaller litters and a higher probability of cub loss than multiparous females [15, 33], which was partially supported by this study. However, the current sample size is not sufficient to draw conclusions on this issue.

**Table 4:** Summary of reproductive records for each female brown bear in the Rusha area, Shiretoko Peninsula, Hokkaido, Japan, 2006–2016.

Bear ID	Age of primiparity	No. of reproductive events	No. of reproductive intervals				Total number of cubs	Average litter size
			1yr	2yr	3yr	4yr		
KR		5		4			10	2.00
WK		4		2	1		7	1.75
DR	5	3		1	1		5	1.67
BE		4		2	1		9	2.25
PK	5	2		1			4	2.00
RI		3		1		1	5	1.67
WM		4	1		1	1	7	1.75
LI	5^#^	6	1	4			10	1.67
DC		3		2			6	2.00
KB		4		2			7	1.75
KS		3		2			3	1.00
HC	5	3		2			4	1.33
GB	6	1					2	2.00
GI	5	1					2	2.00
BK	6^§^	0					–	–
Mean/Total	5.3 ± 0.2^¶^	46^†^	2^†^	23^†^	4^†^	2^†^	81^†^	1.76 ± 0.08^¶^
Surviving Litter*				15^†^	4^†^	2^†^		

^*^Intervals between litters in which ≥ 1 cub survived at least 1 year.

^#^The bear had been known to produce cubs at 5 years old according to a previous survey.

^§^The bear was treated as having produced at 6 years old.

^¶^Mean ± SE

^†^Total value

#### Litter size

We observed 81 cubs in 46 litters from 14 bears (Tables 2 and 4). Litter size ranged from one to three cubs, and averaged 1.76 ± 0.08 (SE) cubs/litter (95% CI = 1.61–1.91). The proportions of litters with one, two, and three cubs were 0.30, 0.63, and 0.07, respectively. It must be taken into account that the mean litter size was potentially biased to the lower end of the spectrum, because bears may have lost cubs before the first visual observation. The date of first observation of females with cubs ranged from May 23 to November 11, with July 5 as the average date (i.e., approximately 5 months after birth). We compared the mean litter size observed prior to July 5 ($\bar{x}$ = 1.76, SE = 0.11, *n* = 33) with that after July 5 ($\bar{x}$ = 1.77, SE = 0.11, *n* = 13), but detected no significant differences (*p* > 0.05, Mann-Whitney U test). This suggests that the current result was not underestimated due to the involvement of later observations. However, the number of cubs lost during the first 5 months remains unknown. Mano and Tsubota [6] estimated mean litter size for Hokkaido brown bears, based on hunted bears with cubs from February to May, as 1.8 ± 0.4 (SD), where the data were restricted to females > 6 years old to eliminate the potential influence of primiparous females. In addition, anatomical approaches based on the number of placental scars in the uterine horns, an indicator of implantation rate, estimated mean litter size as 1.76 ± 0.56 (SD) [10], 1.69 ± 0.60 (SD) [12] and 1.91 ± 0.74 (SD) [8] in Hokkaido brown bears. These estimates are comparable to our result, suggesting that cub mortality between birth and early summer (June to July) did not impact the study results.

#### Inter-birth intervals

The reproductive histories of individual bears are shown in Table 2. We observed a total of 31 completed inter-birth intervals (range: 1–4 years) for 14 females (Tables 2 and 4). The proportions of inter-birth intervals of 1, 2, 3, and 4 years were 0.06, 0.74, 0.13, and 0.06, respectively. Cub production in consecutive years (i.e., one-year interval) was observed in two cases, in which the litter loss in the previous year occurred in early July and mid-August. This phenomenon was also reported in other brown bear populations [25, 34, 35]. In the Rusha area, copulatory behavior was reported in October [36], outside of the normal breeding season for brown bears (late spring to early summer [37]), which may have led to the consecutive births.

When limited to 21 completed inter-birth intervals in which ≥ 1 cub survived at least 1 year, the inter-birth intervals ranged from 2 to 4 years, and the proportions of inter-birth intervals of 2, 3, and 4 years were 0.71, 0.19, and 0.10, respectively (Table 4). Successful family breakup was observed in 26 cases. In most cases, the family breakup was completed by the time of the first observation of the year, but six cases were recognized to occur between the second half of May and the first half of June, similar to a previous study in another population [29]. In one case, the two cubs of a litter were weaned in different years, one at 2 years and the other at 3 years old (Table 2).

To the best of our knowledge, this is the first study to calculate inter-birth intervals based on long-term monitoring of Hokkaido brown bears. The inter-birth interval was estimated as 2.43 (Table 5; 95% CI = 2.16–2.76) and 2.53 (Table 5; 95% CI = 2.26–2.85) years in all litters and in litters that survived their first year, respectively. It is noteworthy that the former estimates are potentially skewed higher than they should be, because we might have missed litter losses that occurred approximately 5 months after birth in some cases (e.g., bear DR in 2016; Table 2). In addition, five cases of 2-year intervals were excluded from the later estimates because we could not confirm that the cubs were alive in the second year, which might bias the later estimates. Therefore, the estimates should be treated as the maximum value. In most cases, bears showed 2- or 3-year intervals, but one female (KB) did not produce any cubs for a fourth consecutive year. This bear was known to produce cubs in 1994, indicating that she was ≥ 23 years old as of 2012, the last year of cub production. This infertility may be attributed to reproductive senescence, which is known to occur in the late 20’s in brown bears [38].

**Table 5:** Calculation of the average inter-birth interval between litters for female brown bears in the Rusha area, Shiretoko Peninsula, Hokkaido, Japan, 2006–2016. This estimation included not only completed inter-birth intervals, but also intervals that remained open when the female was last observed [25].

Time period since last litter (yr)	No. periods observed	No. periods ending in cub production	No. incomplete periods not observed the next year	Observed periods ending in cub production (%)	% of all periods available to end in cub production	% of all available periods ending in cub production	Interval length weighted by % producing
*for all litters*							
1	41	2	5	4.9	100.0	4.9	0.05
2	34	23	4	67.6	95.1	64.3	1.28
3	7	4	0	57.1	30.8	17.6	0.53
4	3	2	1*	66.7	13.2	8.8	0.35
Sum	41	31	10			95.6	2.43^§^
*for surviving litters*^*#*^							
1	28	0	3	0.0	100.0	0.0	0.00
2	25	15	4	60.0	100.0	60.0	1.20
3	6	4	0	66.7	40.0	26.7	0.80
4	2	2	0	100.0	13.3	13.3	0.53
Sum	28	21	7			100	2.53

*The female was assumed to give birth in the next (i.e., 5th) year, and the interval length weighted by % producing (0.22; 5 × 4.4%) was added to the final value^§^

^#^ The data included litters that survived their first year.

#### Reproductive rate

Reproductive rate (young born/year/reproductive adult female) varied slightly by method of calculation. Based on the first method (dividing the mean litter size by the mean litter interval), reproductive rate was estimated as 0.72 (95% CI = 0.62–0.85). For the second method (dividing the number of cubs by the number of adult bear-years), for bears ≥ 4 years old (i.e., the potential age of first reproduction in wild brown bears [4, 6]), we observed 81 cubs over 116 bear-years, and consequently, the reproductive rate was estimated as 0.70 (95% CI = 0.53–0.87). For bears ≥ 5 years old (i.e., the earliest age of first reproduction in the Rusha area), we observed 81 cubs over 107–109 bear-years, and consequently, the reproductive rate was estimated as 0.74 (95% CI = 0.57–0.92) to 0.76 (95% CI = 0.59–0.94). Kohira [16] estimated the reproductive rate of brown bears on the Shiretoko peninsula, including some bears in the Rusha area, as 0.60 from 1990 to 2004, which was about 14–19% lower than the current result. Despite the different estimation methods among studies, this discrepancy was attributed to the lower litter size (1.59) in the previous report. It is conceivable that the previous study included a higher rate of late observations (i.e., after cub loss had occurred), although we cannot exclude the possibility of regional differences and/or temporal changes in productivity.

### Estimation of cub survival rates

Among 78 cubs observed between 2006 and 2015, 21 cubs from 18 litters disappeared in the first year, 47 cubs were confirmed as alive in the second year, and 10 cubs were not observed in or after the second year (Table 6), i.e., cub survival rate between 0.5 and 1.5 years old ranged from 60% (worst-case scenario) to 73% (best-case scenario). The bimonthly cub survival rate from June to November, calculated by the Kaplan-Meier technique, is shown in Table 7. The cumulative cub survival rate from June to November was 63% (95% CI = 53–74%). We note that these estimates are not equivalent to the first-year survival rate, because mortality during the first 5 months was not measured. In some brown bear populations, most cub mortality occurs in the pre-breeding and breeding seasons from April to June, mainly due to infanticide by adult males [5, 27, 33–35, 39]. Sexually selected infanticide is considered to be an adaptive male strategy to shorten the available time for mating, by killing the unrelated, dependent offspring of females [40]. However, as previously discussed, litter size in the current study is comparable to those based on the number of placental scars (i.e., implantation rate) [8, 10, 12], suggesting that cub mortality rate in spring is low in Hokkaido brown bears. In addition, most cub disappearances occurred in July and August, i.e., outside of the breeding season. These observations further suggest that cub mortality is mainly due to poor nutrition in the summer, rather than infanticide by adult males in the Rusha area. This is consistent with some brown bear populations in North America where sexually selected infanticide was not considered to be a major cause of cub mortality [28, 41]. However, we do not deny sexually selected infanticide as a potential cause for cub mortality in the Rusha area, because the two cases of consecutive births could have been attributed to it. In autumn, in contrast, only one cub disappeared between early September and late November, which may be attributed to the quick improvement of the nutritional status of mother and cubs after Pacific salmon spawning begins in late August [14].

**Table 6:** Cub survival rate between 0.5 and 1.5 years old in the Rusha area, Shiretoko Peninsula, Hokkaido, Japan, 2006–2015.

Year	No. females with cubs	No. cubs observed	No. cubs survived	Survival rate
2006	3	5	2–5	40–100%
2007	3	5	4	80%
2008	2	3	3	100%
2009	5	9	3–4	33–44%
2010	5	10	9–10	90–100%
2011	5	9	9	100%
2012	5	7	1–3	14–43%
2013	3	5	4	80%
2014	8	17	11–13	65–76%
2015	4	8	1–2	13–25%
Total	43	78	47–57	60–73%

**Table 7:** Kaplan-Meier cumulative survival rate for cubs from June to November, in the Rusha area, Shiretoko Peninsula, Hokkaido, Japan, 2006–2016.

Period	No. at risk	No. of deaths	No. censored	No. new added	Bimonthly survival rate	Cumulative survival rate	95% CI
Jun 1st*	4			26	1.00	1.00	1.00–1.00
Jun 2nd^#^	30	2		15	0.93	0.93	0.85–1.00
Jul 1st*	43	5	1	18	0.88	0.82	0.72–0.93
Jul 2nd^#^	55	2		6	0.96	0.79	0.70–0.89
Aug 1st*	59	8			0.86	0.69	0.59–0.79
Aug 2nd^#^	51	3	1		0.94	0.65	0.54–0.75
Sep 1st*	47	1	4	8	0.98	0.63	0.52–0.74
Sep 2nd^#^	50				1.00	0.63	0.53–0.74
Oct 1st*	50		0		1.00	0.63	0.53–0.74
Oct 2nd^#^	50		1		1.00	0.63	0.53–0.74
Nov 1st*	49		1	4	1.00	0.63	0.52–0.74
Nov 2nd^#^	52		5		1.00	0.63	0.53–0.74

*First half

^#^Second half

It appears that cub survival rate varies depending on the year, although the number of monitored litters in each year was very limited in this study (Table 6). This is likely due to the annual variation in food availability, especially from July to August, the period of highest cub mortality. Although the feeding habits of the bears in this area have not been investigated in detail, availability of calorie-rich food items in the summer, including the seeds of the Japanese stone pine (*P*. *pumila*) and the pink salmon, might affect the cub survival rate. Further study is needed to investigate the relationships between annual variations in food availability and cub survival in the Rusha area.

### Comparison of reproductive parameters with other populations

All reproductive parameters observed in the Rusha area on the Shiretoko Peninsula fell within the range reported in Europe and North America (summarized in [3, 7, 17, 32, 42–45]), and were among the lowest or shortest age of primiparity, litter size, and inter-birth intervals, and ranked at a high level for reproductive rate. The mean age of primiparity and inter-birth intervals were comparable to those in European populations, especially in Sweden [4], whereas the mean litter size was approximately 0.5 cubs/litter lower than European populations and was comparable to some populations in North America [43]. Mean litter size has been correlated with adult female body weight [43–45] and latitude [42], i.e., heavier bears at higher latitudes tend to have larger litters, and vice versa. The Hokkaido brown bear population is at the southern limit of the species’ range, and mean body weight of adult females on the Shiretoko Peninsula is relatively small (103 kg, *n* = 31 [13]), which is in line with the above theory. It has also been suggested that nutritional status is the primary factor regulating the onset of reproduction and reproductive performance in brown bears [32, 42, 44, 46]. Younger age of primiparity, shorter inter-birth intervals, and relatively high reproductive rate are assumed to be due to the high habitat quality of the Rusha area. The Rusha area is considered to be a natural “ecocenter”, defined by Craighead [18] as an area where highly nutritional food is concentrated during a certain time of the year, and aggregated bears use these resources, due to the occurrence of salmon spawning in autumn. Salmonid fish contain higher digestible energy and protein than other bear food items [47], and the availability of salmon has been suggested to influence habitat quality at both the individual and population levels [48]. In addition to salmon, acorns (*Q*. *crispula)*, containing large quantities of carbohydrates and fats [49], are available from September to November in Hokkaido [50]. It has been suggested that food availability during the autumnal hyperphagia period is particularly important for reproductive success in the following year for bear species [51–53]. We conclude that the existence of annually dependable food sources likely has a positive effect on reproduction in the brown bear in the Rusha area.

## Conclusions

The current study provides detailed information on reproductive parameters and cub survival rate based on long-term monitoring of brown bears in the Rusha Area of the Shiretoko Peninsula, Hokkaido, Japan. It is suggested that Hokkaido brown bears have comparatively high reproductive potential, which is achieved by complementing small litter size with early reproductive maturation and short inter-birth intervals. The demographic parameters obtained from this study will be helpful, for example, for the precise forecast of population trends and for the establishment of suitable upper limits of annual capture numbers, which contributes to the conservation and management of brown bears in Hokkaido, Japan.

## Supporting information

S1 Text Genetic analysis and validation of the accuracy of visual identification. (DOCX)

S1 Table Primer sequences for microsatellite analysis and sex identification used in this study. (XLSX)

S2 Table Months and years when the accuracy of visual identification was validated by genetic analysis for each female brown bear observed from 2009 to 2016. (XLSX)

S1 Fig Variation of facial appearances for adult female bears monitored for the estimation of reproductive parameters. Two-letters on each photo indicate the bear’s ID.
(TIF)

S2 Fig Variation of chest marking shapes for adult female bears monitored for the estimation of reproductive parameters. Two-letters on each photo indicate the bear’s ID. The sizes and shapes were variable; a large bib-like marking (WK, WM, and HC), a small point-like marking (KR and KB), and a V-shaped marking (DR, RI, DC, and GI). The remaining six bears, not shown here, did not have recognizable chest markings.
(TIF)

S3 Fig An example of the consistency of facial characteristics and chest marking shapes over different months and years. The month and year on each photo indicates the period when the photo was taken. A) Facial characteristics for bear KR. One of the characteristics was a black line running along the bridge of the nose (arrow), which had been discernible throughout the study period. B) Chest marking shape for bear RI. She had an asymmetric V-shaped chest marking, which was consistent throughout the study period.
(TIF)
