# Supplementary material for: Reproductive parameters and cub survival of brown bears in the Rusha area of the Shiretoko Peninsula, Hokkaido, Japan
Source: PLoS One. 2017 Apr 25;12(4):e0176251. doi: 10.1371/journal.pone.0176251 (PMC5404773; doi:10.1371/journal.pone.0176251)
Supplement: S1 Text — (DOCX) [file pone.0176251.s001.docx]

# **S1 Text. Genetic analysis and validation of the accuracy of visual identification.**

## **Sample collection and DNA extraction**

*Feces* — Fecal DNA collection began in 2009. Whenever we found fresh stool in the Rusha area, including a sample found on the way to the area (approximately 10 km away), the surface was scrubbed carefully with a cotton swab and stored in 70% ethanol from 2009 to 2014 or by using a flocked swab (Flocked Swab R30, Sugiyama-gen Co., ltd., Tokyo, Japan) and stored in ASL buffer (Qiagen Inc., Tokyo, Japan) from 2015 to 2016. DNA was extracted in a laboratory using the Qiagen QIAamp DNA stool mini kit (Qiagen Inc.), according to the manufacturer’s protocol.

*Hairs* — Hair collection began in 2010. We used traditional hair-trap sampling as a primary sampling method. We set up two traps along two rivers in the Rusha area. The trap was triangular and consisted of an approximately 18-m perimeter of double-stranded barbed wires (each side = 6 m in length) at heights of 40 and 85 cm. At the center, deer internal organs were hung as a lure at a height between 330 and 350 cm so as to not be taken by the bears. Each station was monitored by three automatic cameras (HykeCam SP108-J, Hyke Inc., Asahikawa, Japan) on different sides. The collection of hairs and video data was done at 2-week intervals from mid-June to early November. The hairs were collected with forceps and gloves, and placed in an envelope using an envelope/barb cluster. Each envelope was labeled with the trap identification, date, barb number, and location (upper or lower strand). After collection, barbs and forceps were heated with a gas burner to avoid contamination. Hair was dried and stored at –20°C until further analysis. In addition, we collected hairs found on the trees that were used for tree-rubbing. We found five rubbing trees in the Rusha area, and visited them at 2-week intervals from early June to mid-November. Hair collection was done similar to the hair-trap sampling. If the hair was smeared with pine resin, the sample was stored in 70% ethanol in a 15-mL tube. DNA was extracted in a laboratory using the DNA extractor FM kit (Wako, Osaka, Japan), according to the manufacturer’s protocol.

*Skin samples* — Dart biopsy has been commonly used as a minimally invasive method for collecting samples from wild animals [1-3]. The collection of skin samples by biopsy darting began in 2011. We used a CO_2_ injection rifle (Model J.M.ST; Dan-Inject, Børkop, Denmark) with 1.5-mL dart syringes (Dan-Inject) and 20-mm biopsy needles (BIO20; Dan-Inject). We targeted unfamiliar bears that could not be identified by appearance or whose DNA had never been sampled before. To confirm the accuracy of individual identification based on appearance (see below), the 15 monitored females were sampled using a dart biopsy at least once. Cubs < 8 months of age (i.e., before late September) were not targeted in consideration of their small body size. We shot darts at the hip within 30 m from the target. After the syringe was retrieved, skin tissue was removed from the needle and stored in 70% ethanol in a 1.5-mL tube. DNA was extracted in the laboratory using the DNeasy Blood & Tissue mini kit (Qiagen Inc.), according to the manufacturer’s protocol.

## **Genotyping and parentage analysis**

Twenty-one microsatellite markers and one sex marker, amelogenin, were analyzed, according to previous studies [4-8]. The primers and the combination of the primers used in multiplex PCR assays are shown in Table in S1 Table. The analyses consisted of three phases. First, each sample was tested with the primer mix A, including three loci, to check the DNA quality of each sample. Second, samples that demonstrated clear results in the first phase were further tested with two primer mixes, B and C, including six loci. If a PCR amplification failed at any locus, the sample was excluded from further analysis. The genotype data for nine loci were compared with previously obtained genotypes by using CERVUS ver. 3.0.7 software [9]. When a sample did not match up precisely with any individuals, it was tested with the remaining primer mixes, including 12 microsatellite loci and the sex marker, in a final step. The multiplex PCRs were performed in a total volume of 15 μL, consisting of 1 μL of DNA solution, 0.075 μL of Kit Mix 1 (Multiplex Assay Kit; Takara Bio Inc., Shiga, japan), 7.5 μL of Kit Mix 2, 0.5 μL of primer mix (0.25 μM each), and 5.925 μL of PCR-grade water. The mixture was heated to 94°C for 30 s, followed by 40 cycles of 30 s at 94°C, 1 min at 55°C, and 1 min at 72°C, with a final 10 min at 72°C. One microliter of product was diluted in 60 μL (for fecal and hair DNA) or 120 μL (for skin tissue DNA) of distilled water, and 1 μL of this diluted solution was mixed with 0.125 μL of GeneScan 500 LIZ size standard (Life Technologies Japan Ltd., Tokyo, Japan) and 10 μL of Hi-Di formamide (Life Technologies Japan Ltd.). After denaturation at 95°C for 3 min and cooling on ice, each sample was analyzed with the ABI PRISM 310 genetic analyzer (Life Technologies Japan Ltd.). The allele size was determined using GeneScan ver. 4.1 software (Life Technologies Japan Ltd.). When a PCR amplification was weak for any locus, a single PCR was performed with the same primer. Each individual was genotyped for all loci at least twice.

The parentage analysis was performed using CERVUS version 3.0.7 software, based on the maximum likelihood method. This assisted the confirmation of a blood relationship between a mother and dependent young, and also confirmed that young bears were alive following separation from their mothers.

## **Validation of the accuracy of visual identification of bears by genetic analysis**

We validated the accuracy of visual identification for 15 female bears that were monitored for the estimation of reproductive parameters by genetic analysis. S2 Table showed the month and year when DNA samples were collected and analyzed for each bear. We collected DNA directly from each bear at least three times in multiple years, and primarily in different months. The direct DNA collection included: 1) fresh feces that were voided during visual observation, 2) hairs that were left during visual observation (e.g., when they rubbed their bodies on a tree), 3) skin samples by dart biopsy, and 4) hairs that were collected during capture. In addition, when DNA of their dependent young was directly sampled using the above methods (i.e., feces, hairs, or skin samples), parentage analysis was performed to ensure the result supported the blood relationships between a visually identified mother and offspring (i.e., indirect validation). We compared the results of visual identification with the results of genetic analysis, and in all cases, except for one bear (KS), both results were in agreement. Although bear KS was visually discriminable from other adult females whenever observed, it was hard to recognize her over multiple years, due to low appearance frequency and a lack of outstanding characteristics. However, her reproductive history could be followed every year from 2011 to 2016, with the support of genetic analysis. Therefore, we included her in the analysis. In addition to the years when her identity was confirmed by genetic analysis (in 2011, 2012, 2014 and 2015; S2 Table) or by ear-tags (KS was captured and ear-tagged in 2015, and was easily discriminable in 2016), her reproductive status was confirmed multiple times by hair-trap survey (i.e., video data with the support of genetic analysis of collected hairs) from 2013 to 2016. Taken together, the accuracy of visual identification of monitored bears was proved with the support of genetic analysis in the latter half of the study period, which further indicated the reliability of the data in the first half.

# **References**

1. Beausoleil RA, Clark JD, Maletzke BT. A long-term evaluation of biopsy darts and DNA to estimate cougar density: An agency-citizen science collaboration. Wildlife Soc B. 2016;40(3):583-92.

2. Beckwitt R, Shea J, Osborne D, Krueger S, Barklow W. A PCR-based method for sex identification in Hippopotamus amphibius. Afr Zool. 2002;37(2):127-30.

3. Hermosilla C, Silva LMR, Kleinertz S, Prieto R, Silva MA, Taubert A. Endoparasite survey of free-swimming baleen whales (*Balaenoptera musculus, B. physalus, B. borealis*) and sperm whales (*Physeter macrocephalus*) using non/minimally invasive methods. Parasitol Res. 2016;115(2):889-96.

4. Ostrander EA, Sprague GF, Rine J. Identification and characterization of dinucleotide repeat (CA)n markers for genetic mapping in dog. Genomics. 1993;16(1):207-13.

5. Paetkau D, Calvert W, Stirling I, Strobeck C. Microsatellite analysis of population structure in Canadian polar bears. Mol Ecol. 1995;4(3):347-54.

6. Paetkau D, Shields GF, Strobeck C. Gene flow between insular, coastal and interior populations of brown bears in Alaska. Mol Ecol. 1998;7(10):1283-92.

7. Taberlet P, Camarra JJ, Griffin S, Uhres E, Hanotte O, Waits LP, et al. Noninvasive genetic tracking of the endangered Pyrenean brown bear population. Mol Ecol. 1997;6(9):869-76.

8. Yamamoto K, Tsubota T, Komatsu T, Katayama A, Murase T, Kita I, et al. Sex identification of Japanese black bear, *Ursus thibetanus japonicus*, by PCR based on amelogenin gene. J Vet Med Sci. 2002;64(6):505-8.

9. Kalinowski ST, Taper ML, Marshall TC. Revising how the computer program CERVUS accommodates genotyping error increases success in paternity assignment. Mol Ecol. 2007;16(5):1099-106.
