# Supplementary figures and images for: Reproductive parameters and cub survival of brown bears in the Rusha area of the Shiretoko Peninsula, Hokkaido, Japan
Source: PLoS One. 2017 Apr 25;12(4):e0176251. doi: 10.1371/journal.pone.0176251 (PMC5404773; doi:10.1371/journal.pone.0176251)

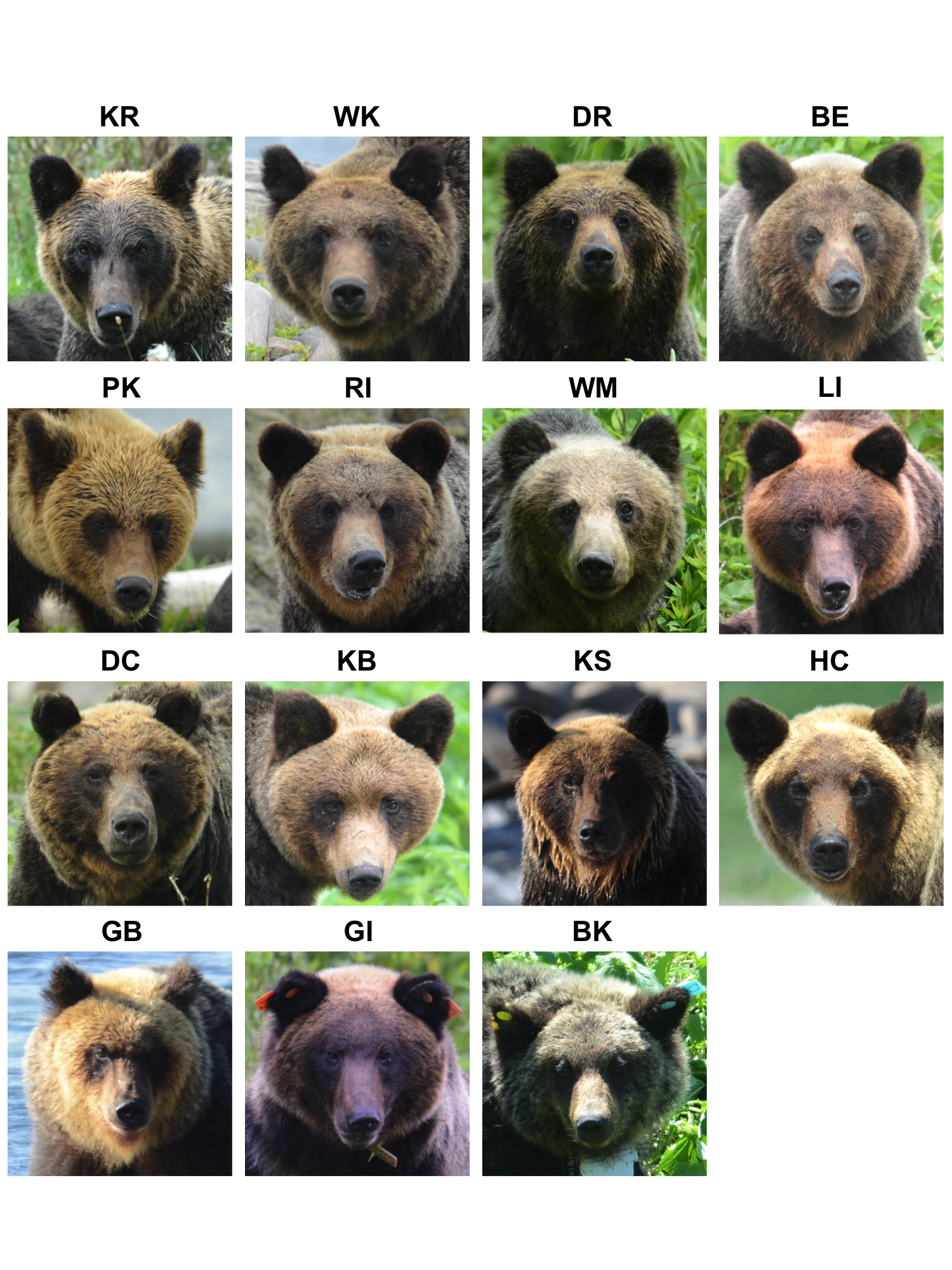

Supplement: S1 Fig — Two-letters on each photo indicate the bear’s ID. (TIF) [file pone.0176251.s004.tif]

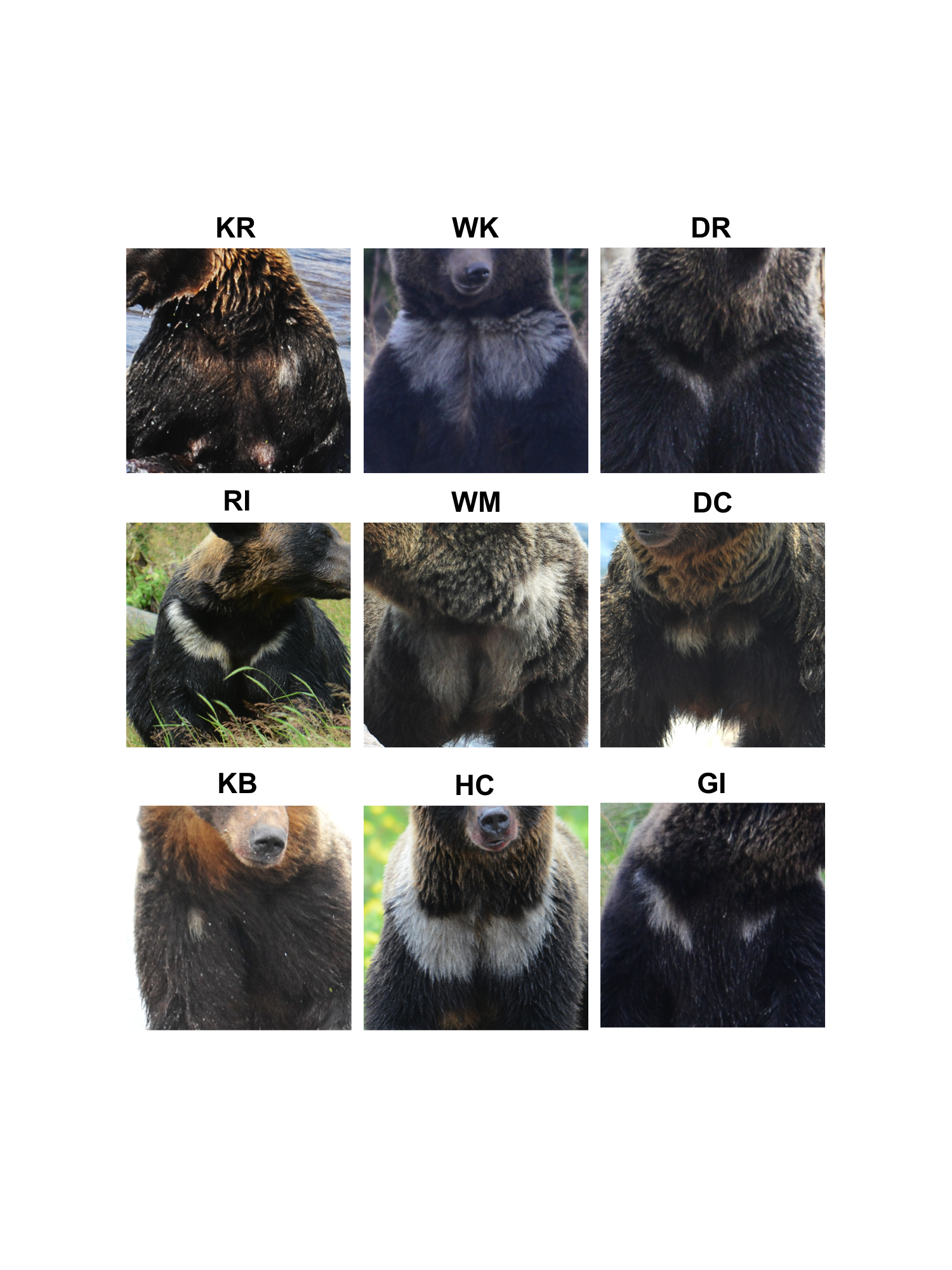

Supplement: S2 Fig — Two-letters on each photo indicate the bear’s ID. The sizes and shapes were variable; a large bib-like marking (WK, WM, and HC), a small point-like marking (KR and KB), and a V-shaped marking (DR, RI, DC, and GI). The remaining six bears, not shown here, did not have recognizable chest markings. (TIF) [file pone.0176251.s005.tif]

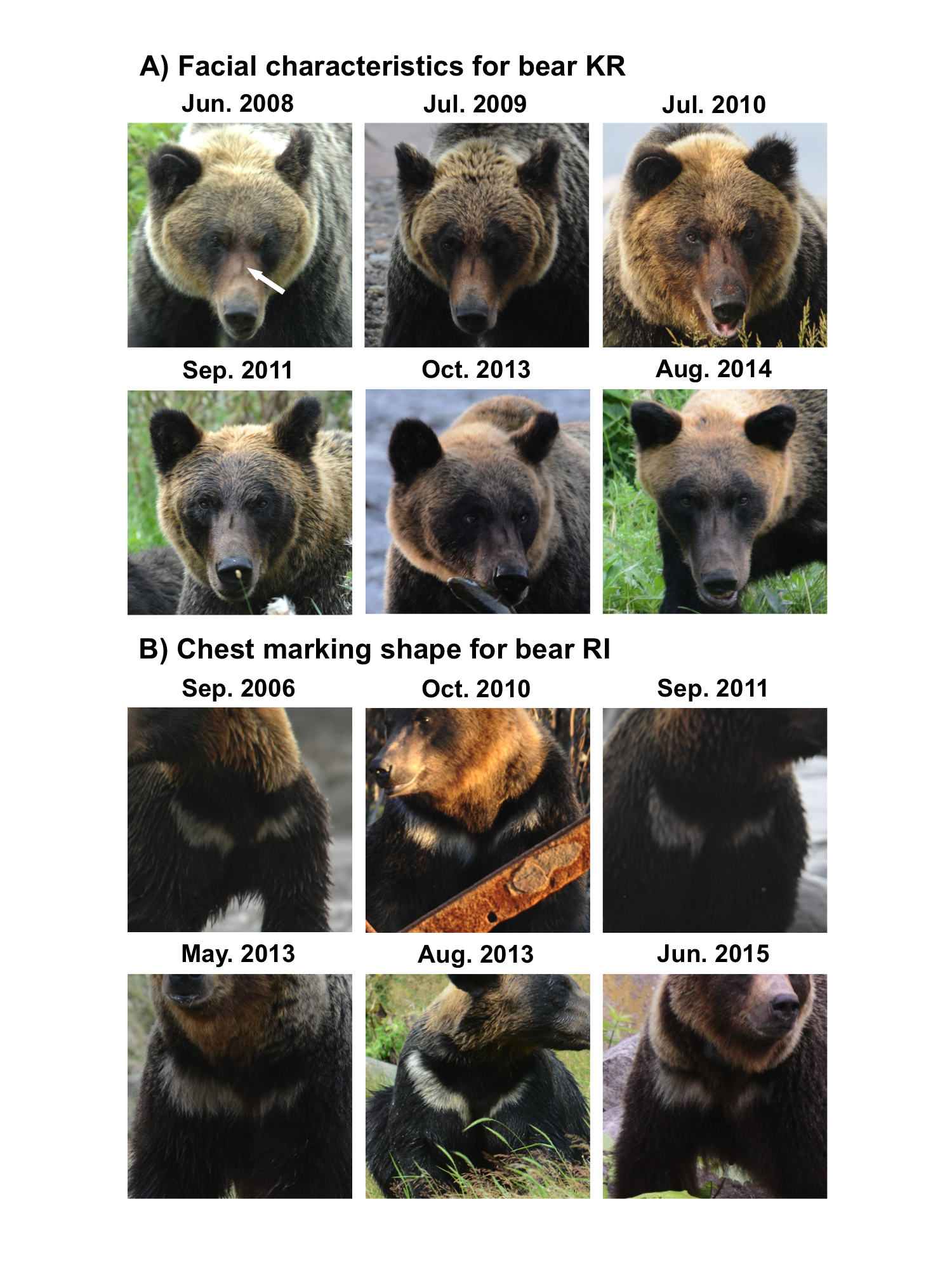

Supplement: S3 Fig — The month and year on each photo indicates the period when the photo was taken. A) Facial characteristics for bear KR. One of the characteristics was a black line running along the bridge of the nose (arrow), which had been discernible throughout the study period. B) Chest marking shape for bear RI. She had an asymmetric V-shaped chest marking, which was consistent throughout the study period. (TIF) [file pone.0176251.s006.tif]
